# *De Novo* SNP Discovery in the Scandinavian Brown Bear (*Ursus arctos*)

**DOI:** 10.1371/journal.pone.0081012

**Published:** 2013-11-18

**Authors:** Anita J. Norman, Nathaniel R. Street, Göran Spong

**Affiliations:** 1 Department of Wildlife, Fish and Environmental Studies, Molecular Ecology Research Group, Swedish University of Agricultural Sciences, Umeå, Sweden; 2 Department of Plant Physiology, Umeå Plant Science Centre, Umeå University, Umeå, Sweden; University of Florence, Italy

## Abstract

Information about relatedness between individuals in wild populations is advantageous when studying evolutionary, behavioural and ecological processes. Genomic data can be used to determine relatedness between individuals either when no prior knowledge exists or to confirm suspected relatedness. Here we present a set of 96 SNPs suitable for inferring relatedness for brown bears (*Ursus arctos*) within Scandinavia. We sequenced reduced representation libraries from nine individuals throughout the geographic range. With consensus reads containing putative SNPs, we applied strict filtering criteria with the aim of finding only high-quality, highly-informative SNPs. We tested 150 putative SNPs of which 96% were validated on a panel of 68 individuals. Ninety-six of the validated SNPs with the highest minor allele frequency were selected. The final SNP panel includes four mitochondrial markers, two monomorphic Y-chromosome sex-determination markers, three X-chromosome SNPs and 87 autosomal SNPs. From our validation sample panel, we identified two previously known parent-offspring dyads with reasonable accuracy. This panel of SNPs is a promising tool for inferring relatedness in the brown bear population in Scandinavia.

## Introduction

Genomic data are useful for understanding wild populations, particularly for wide-ranging and elusive species like the brown bear (*Ursus arctos*). Among many uses, genomic markers can help determine genetic relatedness between individuals in a population, which is key for determining many evolutionary, behavioural or ecological processes [1]. For example, although maternity can often be reliably inferred based on behavioural patterns alone (cf. [2]), assigning paternity is typically more problematic. This is the case for some species that appear to have a monogamous mating system when observed in the wild, yet genetic analyses reveal extra-pair paternity as being common [3]. Detecting paternity can help determine, for example, factors affecting reproductive success (e.g. [4]). In addition, relatedness measures can be used to detect hybridization events or identify introgression zones (e.g. [5,6]). Detecting inbreeding can be critical for small or reintroduced populations that are prone to inbreeding depression [7]. Genetic relatedness can also be used to measure gene flow and uncover dispersal patterns [8]. As such, the use of high quality genomic markers can enhance our understanding of biological processes in wild systems as shown by relatedness studies on *Ursus* species (e.g. [9–12]).

Resolving relationships in wild populations can be challenging [13] and is typically reliant upon high quality markers with high genomic resolution [14]. Insufficient genomic resolution (either too few markers or unequal representation throughout the genome) can result in inflated genotypic variances and, thus, lower confidence making relatedness inferences problematic. Single nucleotide polymorphisms (SNPs) occur frequently throughout the genome rendering them suitable for analyses requiring high genomic resolution. In addition, some marker types (e.g. microsatellites) are error prone and suffer from technical artifacts such as null alleles. Erroneous genotypes can cause significant biases in genetic monitoring [15]. The bi-allelic nature of SNPs leads to simplified genotyping that is less erroneous [16].

Until recently, genome-wide SNP marker development was prohibitively expensive and time-consuming. With the advent of next-generation sequencing (NGS) technologies, SNP development has become more accessible. Correspondingly, SNPs are increasingly being utilized in studies of non-model organisms (e.g. [17–22]). For example, Miller et. al. [23] developed a set of 100 SNPs for polar bear (*Ursus maritimus*) and brown bear to investigate phylogenetic history. However, processing the vast amount of data generated by NGS technologies has become a significant challenge due to the large demand for bioinformatics expertise, computational load and data storage infrastructure [24]. Therefore, methodologies that reduce the necessary amount of data and computational complexity within the limits of the study can simplify the complex downstream analyses and reduce demands on infrastructure. For example, application of a reduced representation libraries approach (RRL) for SNP discovery [25] considerably decreases the amount of sequencing data required while simultaneously allowing for high genomic resolution. Advances to the RRL methodology have recently been developed that further increase its utility (e.g. [26]). 

An informative SNP panel is one in which each SNP maximizes the differences in allelic representation across individuals within a population when compared to all other SNPs. Hence, SNPs with higher minor allele frequencies (MAF) and that are not in linkage with each other are more informative for relatedness inference [27]. The number of SNPs required for making reliable relatedness estimates has been debated (e.g. [28,29]). However, depending on population characteristics, sample size and level of marker informativeness, there is evidence that relatedness inferences can be reliably inferred using a minimum of 60 SNPs [27]. NGS-based methods have enabled detection of thousands to hundreds of thousands of SNPs (depending on the species, proportion of the genome sequenced and read depth), representing orders of magnitude greater than what is required for relatedness studies. Thus, data reduction through the use of RRL approaches and the application of highly stringent filtering criteria to retain only the highest quality, informative SNPs is particularly relevant.

Historically, over-hunting and habitat fragmentation have negatively affected many brown bear populations, a trend that led to the loss of much of the historical geographic range in Europe [30]. Currently, Scandinavia is among the few regions where the brown bear population is increasing [31]. Maternally-inherited mitochondrial DNA from the control region have shown that the brown bear population in Sweden and Norway consists of two distinct lineages with more than 7% differentiation between them; the eastern European lineage situated in the north, and the western European lineage situated in the south-central part of Sweden [30]. The southern population is of particular conservation interest since it is one of the few relic populations of the western European lineage [30].

In this study, we developed *de novo* a set of 96 high quality SNPs by applying an NGS-based RRL approach with an ascertainment panel of brown bears across the geographic range in Scandinavia. A SNP-chip was designed primarily to facilitate relatedness studies, although it can be useful for a wider range of studies. In addition to autosomal SNPs and Y-chromosome sex-determination markers, we included mitochondrial (mtDNA) and X-chromosome SNPs to further facilitate determination of parental ancestry. Our approach to reducing data complexity allowed for efficient and simplified ascertainment of a medium-throughput panel of highly informative SNPs. 

## Materials and Methods

### Sample Collection and DNA Extraction

**Figure 1:**
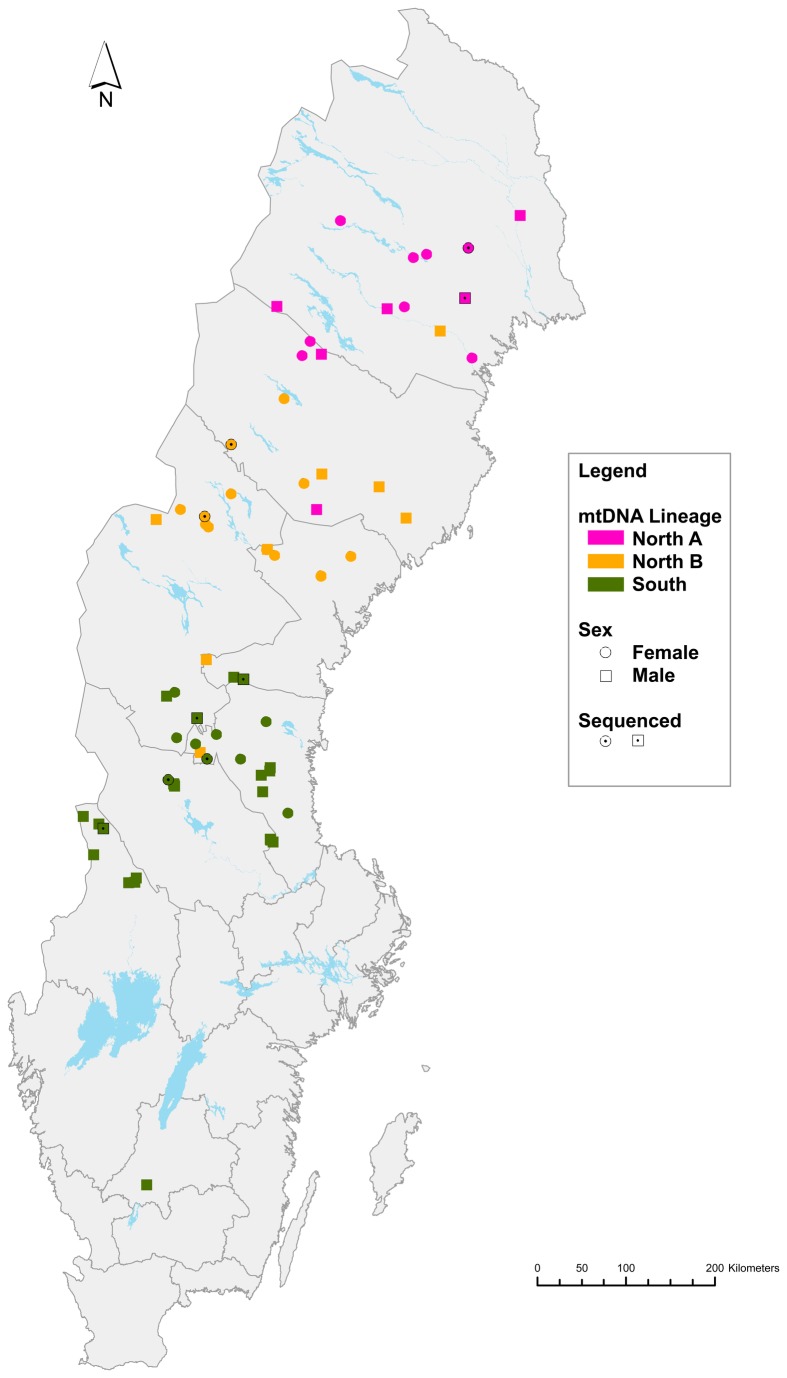
Brown Bear Sampling Locations. Points represent sampling locations for each individual used in the validation genotyping (n=68). They are graphically presented to indicate the mitochondrial-based lineage the individual belongs to, whether it is female or male, and the nine individuals that were initially sequenced.

We obtained 68 samples from the National Veterinary Institute (Statens veterinärmedicinska anstalt (SVA), Uppsala, Sweden) from bears deceased either through a licensed hunt or that were found dead through other causes (e.g. natural mortality, vehicle/train collisions). No bears were killed for the purpose of this study or for other research endeavours. Samples were obtained with full consent by SVA. Samples were chosen to represent an even sex ratio and the full geographic range of brown bear throughout Sweden (Figure 1). The majority of samples (n=56) were collected from muscle tissue, while others were from liver (n=10) and skin (n=2). Samples were collected between 2000 and 2012 and, except for liver, were stored in ethanol prior to DNA extraction. Liver samples were kept frozen at -20°C. 

DNA was extracted using the QIAsymphony SP and the QIAsymphony DNA kit (Qiagen, Hilden, Germany) according to manufacturer’s instructions. Nucleotide quantity and purity were assessed using a spectrophotometer (NanoDrop, Thermo Fisher Scientific, Massachusetts, USA). DNA quality for extractions used for sequencing was visually assessed by gel electrophoresis using the Kodak Electrophoresis Documentation and Analysis System 120 (Eastman Kodak Company, Rochester, USA).

### DNA Sequencing

We used a combination of targeted and anonymous sequencing approaches to identify SNPs that are informative of parental lineage and that are autosomal respectively. The targeted approach involved mitochondria and the Y-chromosome, while the anonymous approach involved high-throughput sequencing of reduced representation libraries.

#### Mitochondrial DNA

Four primer pairs were designed from the published mitochondrial genome (NCBI Accession # EU497665.1) of a European brown bear using Primer3 [32]. Each primer set was designed to amplify a product of approximately 500 base pairs (bp) (Table 1).

**Table 1:** Mitochondrial DNA primer pairs for brown bear (*Ursus arctos*) based on the mitochondrial genome #EU497665.1 (NCBI, Bethesda, USA).

Primer Name	Primer Position**	Product Length***	H/L§	Primer Sequence (5’-3’)
Urs_mtDNA.45H	4542	546	H	CCCATTATCACAGCAAGCATT
Urs_mtDNA.50L	5087	546	L	GCAATGGTGATTACGGTTGAT
Urs_mtDNA.66H*	6661	501	H	GCACCTAGCAGGCATCTCTT
Urs_mtDNA.71L*	7161	501	L	CCTGTCGGGATAGCAATGAT
Urs_mtDNA.94H*	9422	499	H	GTTCGCTGTAGCCCTCATTC
Urs_mtDNA.99L*	9920	499	L	ACACTCCGGATGCAAGAAGT
Urs_mtDNA.134H	13497	499	H	CCTGTGCTCTCACCCAGAAT
Urs_mtDNA.139L	13995	499	L	CGCTTGATGGAATTGATTAGG
Urs_mtDNA.30H	3092	468	H	TTCCTTCCATGAGCTAGCAA
Urs_mtDNA.35L	3559	468	L	GCTCTGCCACCCTAACAAAG
Urs_mtDNA.145H*	14562	487	H	CGAATCCCCCGTATCATAAA
Urs_mtDNA.150L*	15048	487	L	TCGGATGTTGGTCATTAAGGT
Urs_mtDNA.155H	15502	508	H	GGAACGGACCTGGTAGAATG
Urs_mtDNA.160L	16009	508	L	AAAATAGGCATTGGCTTAGGG
Urs_mtDNA.160H	16083	529	H	CGGACAACTAGCCTCCATTC
Urs_mtDNA.166L	16611	529	L	GGAGCGAGAAGAGGTACACG

* Markers from these sequences included in final SNP set

** Position according to accession # EU497665.1 (NCBI)

*** Includes primers

^§^ Heavy (H) and light (L) strands

Each of ten samples were PCR amplified in a total reaction volume of 20 μl consisting of 2.5μl, 10-40ng/μl DNA, 0.5μl 10μM each of the forward and reverse primer, 12.23μl distilled water, 0.5μl 2.5mM dNTP’s, 2.0μl 10X *Taq* buffer*, 1.6μl 2.0mM MgCl_2_* and 0.17μl *Taq* DNA polymerase* (*Fermentas *Taq* DNA Polymerase – native). The optimized PCR conditions for all primer pairs include 1 cycle of 94°C for 3 min; 20 cycles of 94°C for 20 s, 60°C less 0.5°C/cycle for 30 s, 72°C for 30 s; 15 cycles of 94°C for 20 s, 50°C for 30 s; and 72°C for 5 min. PCR amplification was confirmed through gel electrophoresis. The remainder of the product was Sanger sequenced by Medicinsk och klinisk genetik (Norrlands Universitetssjukhus, Umeå, Sweden) on a 3730 xl DNA analyzer (Applied Biosystems, Foster City, USA). Sequences were aligned using BioEdit (v 7.0.9; Tom Hall, Ibis Biosciences, Carlsbad, USA) and manually screened to identify SNPs.

#### Y-Chromosome

Four published Y-chromosome primer pairs were selected (DBY3, DBY5, DBY8 and SMCY7; [33]). The total expected number of base pairs for all four products was 1,550.

Each of 12 samples from males were PCR amplified in a total reaction volume of 20μl consisting of 2.5μl, 10-100 ng/μl DNA, 0.5μl of 10μM each of forward and reverse primer, 12.23μl and 12.63μl distilled water for DBY3, DBY5 and DBY8, SMCY7 respectively, 0.5μl 2.5mM dNTP’s, 2.0μl 10X *Taq* buffer*, 1.6μl and 1.2μl 2.0mM MgCl_2_* for DBY3, DBY5 and DBY8, SMCY7 respectively and 0.17μl *Taq* DNA polymerase* (*Fermentas *Taq* DNA Polymerase – native). The PCR conditions were optimized and the resulting products confirmed, sequenced and processed following the same conditions and steps as for the mtDNA. 

#### Reduced Representation and High-Throughput Sequencing

To determine an appropriate balance between genomic coverage and read depth, we performed preliminary calculations for developing a reduced representation library using BglII (A/GATCT) restriction enzyme, based on [25]. Our calculations were based on two assumptions: That the average fragment length resulting from a BglII digest of the brown bear genome is similar to that of the human genome (~3,100 bp; [25]) and that the genome size of the brown bear is approximately 2.4 Gbp (giga base pairs) based on the measured C-value (2.75 pg; [34]) relative to the dog (2.80 pg) whose genome size is approximately 2.5 Gbp [35]. With this, we estimated that we could obtain a genomic coverage of ~1% with a read depth of ~40X for each sample if we used all genomic fragments between 100 and 700 bp after a BglII digest. After the sequencing was conducted, a draft genome assembly was made available to us (pers. comm. Axel Janke, Senkenberg Institute, Germany) which we used to perform an *in silico* digestion with BglII to test the above assumptions.

We digested 0.5 μg each of ten DNA samples (liver) individually for 16 hours with BglII (Fermentas, Vilnius, Lithuania) according to manufacturer’s instructions. To remove the activated enzyme, samples were purified using the MinElute Reaction Cleanup kit (Qiagen, Hilden, Germany) in two elutions. The second elution was visualized by gel electrophoresis to assess the quality of the digestion. Digested DNA samples were sent to the Science for Life Laboratories (SciLifeLab, Stockholm, Sweden) for library construction and preparation. Fragments 100 to 700 bp were excised and blunt end repaired. Paired-end, multiplexed adapters were ligated to the fragments by sample and equimolar concentrations were measured and sequenced on one lane of Illumina HiSeq2000 resulting in 2x100 bp paired-end reads with insert sizes ranging from zero to 500 bp (mean 249.01 +- 130.06). Sequence data has been submitted to the NCBI Sequence Read Archive (SRA) under the study accession number SRP023544 (http://www.ncbi.nlm.nih.gov/sra/?term=srp023544).

### Quality Filtering and Alignment

Sequenced reads were demultiplexed using the barcode_splitter option of the FASTX Toolkit (v 0.0.13; http://hannonlab.cshl.edu/fastx_toolkit/) and adapters removed with cutadapt (v 0.9.3; [36]). Reads were trimmed to 100 bp, and quality filtered using the FASTX Toolkit trimmer and quality_filter options respectively using the settings: q 10, p 70. Sequence quality was assessed using FastQC (v 0.9; Babraham Bioinformatics, Cambridge, UK) both before and after filtering. After quality filtering, paired reads were synchronized and reads not containing the cut site (GATCT) were removed using customized python scripts. The remaining reads were used as input for analysis and SNP detection using Stacks (v. 0.9995, [37]) with the settings: m 2, M 3, n 1, t and H. Consensus reads generated by Stacks were aligned to the draft genome (see above) using Bowtie 2 (v 2.0.0, [38]) with the settings: q, X 700.

### SNP Calling and Validation

**Figure 2:**
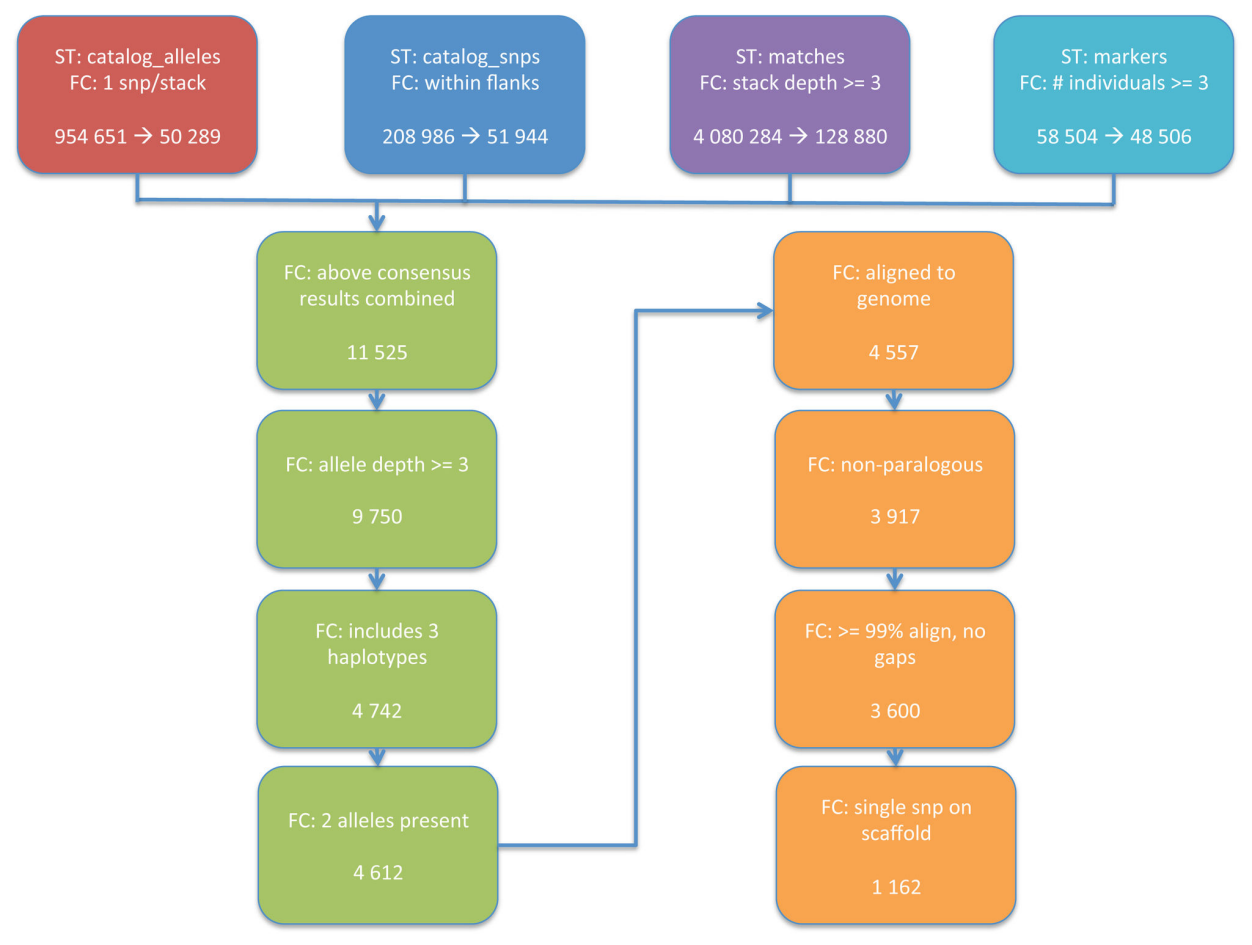
Filtering Criteria Applied to Putative SNPs. Each step of the filtering process and the number of SNPs remaining are shown in sequence. Putative SNPs were identified through Stacks software. The files generated through Stacks were used in the filtering process and are denoted with ST. The first four filtering criteria (FC) were applied in parallel as each file contained different information. The orange boxes indicate filtering criteria that were applied using the software blastn and the draft genome assembly.

The results from Stacks were imported into a custom MySQL (Oracle Corporation, Redwood City, USA) database where, in combination with python scripts, putative SNPs were filtered to remove ones of low quality (Figure 2). First, we ensured that only one SNP could exist on any given read and that the SNP must be at least 20 nt (nucleotides) from the 5’ end and at least 35 nt from the 3’ end of the read. The rationale behind the one SNP per read was to both reduce the number of pseudo SNPs resulting from sequencing error and to eliminate any hypervariable sequences. We required that the SNP be located in the middle of the read to ensure that adequate flanking sequences remained on either side for subsequent SNP assay development. We removed any SNP that appeared in less than three individuals and that did not contain all three genotypes (i.e. aa,ab,bb). This was to allow us to choose higher quality SNPs with greater representation across the individuals. Homology searches against the reference genome draft assembly were then performed using Blastn (NCBI, Bethesda, USA). Since our aim was to develop a 96-well chip, we could afford to be strict in our filtering, therefore we reduced the number of SNPs by choosing only those that aligned with a minimum 99% identity (allowing for one mismatch assumed to be the SNP) and no gaps. We removed reads that aligned multiple times to ensure that we would not end up with pseudo SNPs due to paralogous sequences. Likewise, we chose only SNPs that aligned to scaffolds with no other SNP to minimize linkage between SNPs due to close physical vicinity. Finally, SNPs were manually screened to ensure exclusion of those with homopolymers in the flanking region as well as for adequate allelic representation. A total of 150 SNPs were selected, assays were developed (Fluidigm Corporation, San Francisco, USA) and then used to genotype 68 brown bear samples using the Fluidigm Biomark.

### SNP-Chip Development

Genotyped individuals were analyzed to determine both relatedness using the Graphical Representation of Relationship errors approach [39] and the number of subpopulations using multidimensional scaling (MDS), both as described in [40] and implemented using the R programming language [41]. Where dyads were represented as outliers on a boxplot (0.95 CI) indicating either very close or very distant relatedness, we removed one of the pairs in subsequent analyses. The remaining samples were grouped into three subpopulations based on mitochondrial lineage as confirmed by the MDS analysis. To identify the most informative and highest quality SNPs, we calculated descriptive statistics on the validated SNPs including minor allele frequency (MAF), expected and observed heterozygosity (H_E_ and H_O_), and using Genepop v. 4.2 [42,43], Hardy-Weinberg Equilibrium and F_ST_. SNPs were selected for the final SNP-chip (96 SNPs) if they were among those with the highest MAF. It is important to note that depending on time since divergence, ascertainment bias may affect the utility of these SNPs in brown bear populations outside of Scandinavia. These SNPs were analyzed for linkage disequilibrium with D’ statistics in the R packages genetics v 1.3.8 (http://cran.r-project.org/web/packages/genetics//index.html) and LDheatmap v 0.99 [44]. The 90 nuclear SNPs (excluding mitochondrial and Y-chromosome markers) are published in dbSNP through NCBI (Bethesda, USA) with ss numbers from 778079577 to 778079666.

### Relatedness Analyses

To determine how informative the final set of SNPs would be in assessing relatedness, we conducted two additional analyses. First, we incorporated all autosomal SNPs (n=87) and unrelated individuals (n=50) and ran Structure [45–48] using a burnin of 100,000 and MCMC reps of 500,000 with 20 iterations each of K = 2 to K = 5 and default settings. Second, we calculated the Lynch and Ritland relatedness estimator (r) [49] using Coancestry [50] with all individuals (n=68) to identify dyads with possible first-order relatedness (i.e. parents or full siblings). To exclude possible parent-offspring relationships of all dyads with r > 0.40, we used a customized python script to calculate the number of alleles shared at all loci excluding those dyads that have at least one locus where no alleles are shared. Our sample panel consisted of two known parent-offspring dyads.

## Results and Discussion

### DNA Sequencing

A total of 2015 and 1489 bp were sequenced in the mtDNA and the Y-chromosome respectively. Sequencing of the RRLs generated ~20 Gbp of data from nine samples. One sample failed to sequence for unknown reasons. After quality filtering and removal of reads not containing the restriction cut site, approximately 30 million paired reads (32%) remained. We suspect that the low retention rate is a result of unintended sequencing of degraded DNA as indicated by the gel visualization of the restriction digest. However, 82% of retained reads (unpaired) aligned to the draft genome suggesting that the sequence data used in downstream analysis was of high quality. This is promising for sequencing projects that are dependent on low quality DNA (e.g. ancient DNA or environmental DNA). 

We utilized a draft genome assembly to test our assumptions regarding the cut frequency of the BglII enzyme and the genome size of the brown bear. Table 2 shows the results of an *in silico* digestion of the draft genome (for which the genome size estimate of 2.4 Gbp is in accordance with an independent estimate by Miller et. al. [23]) using the BglII restriction enzyme in comparison with our preliminary calculations. The differences for the two assumptions were minor (5% and 12% respectively), confirming the appropriateness of our approach in ascertaining the right balance between genomic coverage and read depth.

**Table 2:** Comparison of the estimated and actual genomic calculations for a BglII restriction digest of the *Ursus arctos* draft genome.

	Avg Frag Size (d)	Genome Size (G)	# Unique Fragments (D)*	Read Depth Per Individual	Max Genomic Coverage (%)
Estimated	3100	2,400,000,000	131,910	38	1.10
Genome**	3465	2,277,069,268	93,678	53	0.82
% Diff***	-11.8	5.1	29.0	-28.3	25.5

* Includes only genomic fragments between 100 and 700 bp

** Unpublished data (Pers. comm. Axel Janke, Senkenberg Institute, Germany)

*** A minus sign indicates underestimates

### SNP Calling

A total of 57 haploid SNPs were identified from the mtDNA sequences. Fifty-four of these separated the two major maternal haplotypes that distinguish the eastern European lineage from the western European lineage. We chose four of these mtDNA SNPs for lineage identification in the final SNP-chip.

The Y-chromosome sequences showed no variable sites, concurring with the theory that mammalian Y-chromosomes have low levels of nucleotide diversity [51]. While this does not allow for enhanced resolution of data on paternal lineage, it remains useful for sex-determination. We therefore developed two monomorphic “SNPs” based on the Y-chromosome sequences by designing assays around one non-variable nucleotide. 

A total of 1.4M stacks (i.e. consensus sequences generated from sets of co-aligning reads representing restriction products) were created with aligned reads both within and among individuals (n=9). Of these, 105k (14%) contained at least one putative SNP, although this is likely to be an overestimate of the true number of SNPs due to presence of sequencing errors. Mean read depth within each individual ranged from 3X to 8X and is likely to be underestimated due to duplicate stacks resulting from the use of stringent parameters. Although we estimated an expected read depth of 40X, only 32% of the sequence data generated was utilized for creating stacks and, as such, expected depth was reduced to approximately 12X per individual. Nevertheless, read depth was sufficient to reliably call SNPs as shown by our validation results below.

After the initial filtering criteria (i.e. one SNP per read, SNP located in the middle of the read, and representation of all three haplotypes) were applied, 4,612 putative SNPs remained. These SNPs were then further reduced to 1,162 after application of the additional filtering criteria using the draft genome assembly. Figure 2 depicts the filtering process in more detail.

### SNP Validation

**Figure 3:**
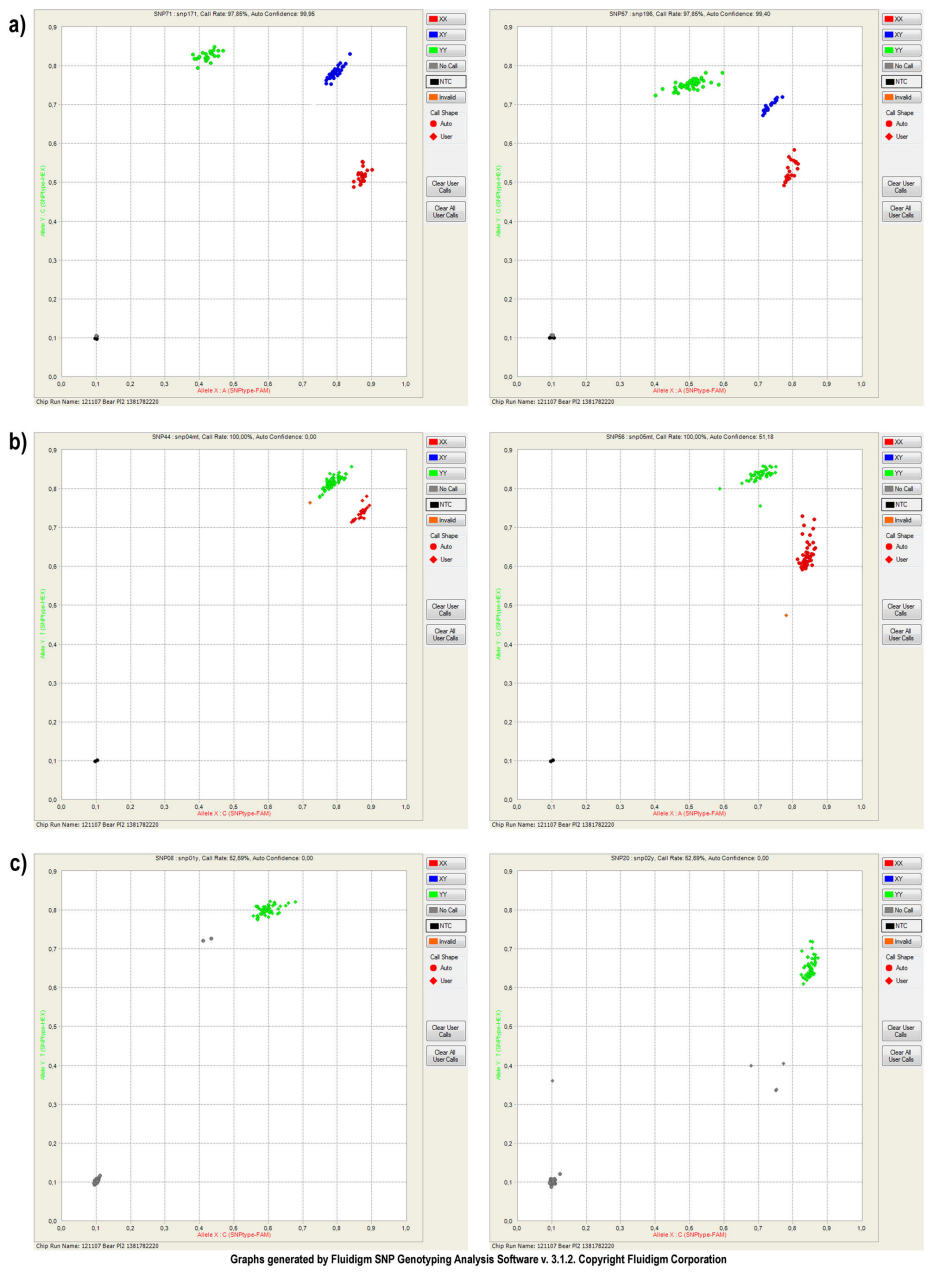
SNP Scatterplots. Scatterplots generated by the Biomark system (Fluidigm, San Francisco, USA) showing allelic clustering based on fluorescence for a) autosomal SNPs, b) mitochondrial haplotype markers, and c) Y-chromosome monomorphic sex-determination markers with male fluorescence.

Our final panel of putative SNPs included 144 nuclear SNPs, four mtDNA markers and two Y-chromosome sex-determination markers. We used a panel of 68 individuals from throughout Sweden (including the initially sequenced individuals) for validation. A total of 144 of the 150 SNPs (96%) produced good results. Of the six that failed, one gave no signal, one was monomorphic, and the remaining four did not pass the control checks. Since we ran two chips with 96 SNPs each, we effectively ran 56 of the SNPs twice as a control. In addition, we included both negative (water in place of DNA) and positive controls. The positive controls included duplication of some of the samples including those that were originally sequenced. The working SNPs passed all of the control checks and we did not detect a single error (error rate < 0.001). Figure 3a shows two representative scatterplots of successful SNPs. 

The four mitochondrial markers and two Y-chromosome markers segregated according to expectations. Since mitochondria are haploid, there is no possibility for heterozygotes to exist. As expected, scatterplots of these SNPs display only two distinct clusters each representing one of the two possible alleles (Figure 3 b). Similarly, since the Y-chromosome markers were intentionally monomorphic, there should be no possibility for either heterozygotes or a second allele and clusters should contain only male samples. The scatterplots indeed show only one cluster (one allele) and contained male samples as verified with demographic data (Figure 3 c).

While it has been documented that there are several subpopulations within the northern population [52], analysis of our original mitochondrial sequences from 10 individuals identified only two haplotypes representing the northern and southern populations. However, the genotyped individuals in the validation run revealed a third haplotype indicating maternal-based substructure within the northern population in concordance with [52]. With our data, we therefore recognize three mitochondrial-based haplotypes: the North A (ABAA), North B (AAAA), and South (BBBB) with Ua03mt, Ua04mt, Ua05mt, Ua07mt markers respectively.

### SNP-Chip Development

**Figure 4:**
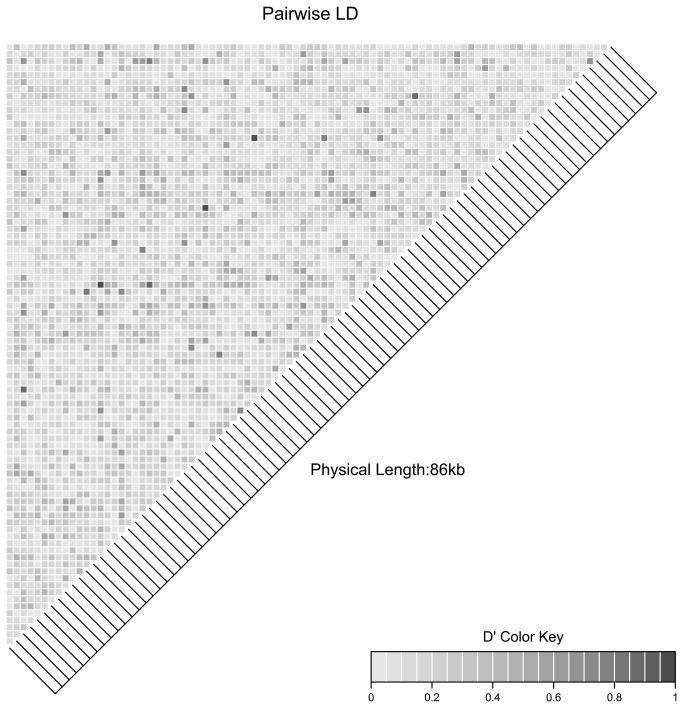
Linkage Disequilibrium Heatmap. Pair-wise linkage disequilibrium (D’) for autosomal SNPs (n=87) with unrelated individuals (n=50).

To reduce the 144 working SNPs to the 96 represented on the chip, we included four mtDNA markers, two Y-chromosome markers and subsequently selected the autosomal SNPs with the highest minor allele frequency (MAF) (valid for the Scandinavian population) and that demonstrated a clear divergence of clusters in the scatterplot. These 96 SNPs were further analyzed for MAF (mean= 0.39), H_E_ and H_O_, HWD and F_ST_ (Table S1). After removing outliers (n=18) based on close or distant relatedness (see methods) and sorting into subpopulations by mitochondrial lineage, seven SNPs remained significant, but only within one of the three subpopulations for Hardy-Weinberg disequilibrium (HWD). A linkage disequilibrium analysis (Figure 4) revealed that two pairs of SNPs were linked (D’ = 0.9996, 0.9411 respectively). It is likely that there are more pairs that are in high linkage disequilibrium, as would be expected when there are less chromosomes than SNPs [28]. However, further investigation using the draft genome assembly and the pairs of SNPs with high D’ values revealed that these two pairs of SNPs were found to be in close proximity to each other on neighboring scaffolds, thereby confirming linkage. However, the integrated fluid circuit of the Fluidigm Biomark (Fluidigm Corporation, San Francisco, USA) is not prespotted allowing for easy replacement of individual SNP assays by any lab operating the Biomark. Future configurations thus allow for the replacement of one SNP per linked pair with an unlinked SNP.

We determined that three of the 96 SNPs most likely occur on the X-chromosome. In all three cases, all male samples (n=36) were homozygous for the same allele whereas female samples (n=32) were either homozygous or heterozygous. The chance of a Type I error (i.e. all 36 males appearing as homozygotes by chance) in inducing loci that are on the X-chromosome with 36 male samples and a MAF of 0.31 (our lowest MAF for X-chromosome SNP) is one in 535 million. We therefore feel confident in stating that these SNPs occur on the X-chromosome. These SNPs will be advantageous when determining parentage by allowing additional exclusion power in cases where alleles are not in concordance with putative parent-offspring pairs. 

### Relatedness

**Figure 5:**
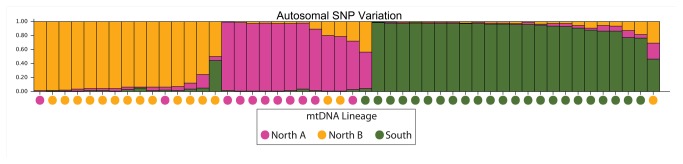
Inferred Population Structure. Inferred population structure based on autosomal SNPs using Structure (burnin period 100,000 cycles; 500,000 MCMC reps) with K=3, sorted by Q. The coloured circles below each bar represent the mitochondrial haplotype. The colour was chosen based on the bar plot colours where the majority of the mitochondrial lineage (North A, North B, South) is found.

The population structure analysis based on Structure [45–48] resulted in K = 3 subpopulations as the most likely scenario (Figure 5) based on the ln probability of data being the lowest of all Ks as described in the documentation. The three mitochondrial haplotypes (North A, North B and South) are well matched to the three autosomal-based subpopulations with only six individuals of 50 having mismatching haplotypes when compared with the individuals’ major population assignment for Structure results. 

To assess the performance of the SNP set to determine a minimum of first-order relatedness (i.e. parent-offspring or full siblings; r=0.50), we calculated the Lynch-Ritland [49] relatedness estimator (r). We filtered for all dyads whose values were greater than 0.40 (n=18; Table 3). Among these were two known parent-offspring dyads from our sample set (r = 0.6176, 0.4922). In addition, we were able to exclude parent-offspring relatedness in one dyad (r = 0.5725) based on X-chromosome data. Out of these 18 dyads with possible first-order relatedness, we could exclude 14 as parent-offspring as a result of them having one or more loci with 0 shared alleles (parent-offspring dyads will always have at least one allele in common by descent at all loci). The two known parent-offspring dyads are included in the four that cannot be excluded as parent-offspring thereby confirming accurate genotyping. As we genotype more individuals in a subpopulation, we will obtain more accurate MAFs, which will allow for more precise estimates of relatedness. These results indicate that the SNP set holds much promise for relatedness analyses.

**Table 3:** Pairwise relatedness estimates using the Lynch-Ritland r estimator [49] on all validation samples (n=68) where r> 0.40, thus indicating possible first order relationships.

					Confidence Limits		
Ind_1	Ind_2	Sex	Hap	r_xy_	2.5	97.5	P-O
Ua12	Ua82	M-M	NA	0.44	0.24	0.60	Excluded
Ua13	Ua37	M-F	S	0.44	0.24	0.60	Excluded
Ua13	Ua72	M-M	S	0.41	0.18	0.56	Excluded
Ua19	Ua85	F-M	NA	0.57	0.45	0.73	Excluded
Ua23	Ua89	M-M	S	0.48	0.36	0.59	Possible
Ua40	Ua41	M-M	S	0.41	0.22	0.57	Excluded
Ua42	Ua45	M-F	S	0.46	0.32	0.59	Possible
Ua43	Ua73	M-M	S	0.41	0.24	0.55	Excluded
Ua50	Ua51	M-M	NB	0.54	0.33	0.67	Excluded
Ua67	Ua82	M-M	NA	0.44	0.28	0.59	Excluded
Ua80	Ua91	M-M	NB	0.43	0.26	0.57	Excluded
Ua85	Ua100	M-F	NA	0.49	0.33	0.65	Excluded
Ua88	Ua96	F-F	NB	0.55	0.42	0.69	Excluded
Ua88	Ua97	F-F	NB	0.44	0.24	0.63	Excluded
Ua96	Ua97	F-F	NB	0.42	0.22	0.57	Excluded
Ua96	Ua99	F-F	NB	0.40	0.23	0.58	Excluded
Ua98	Ua99	F-F	NB	0.62	0.51	0.75	Known
Ua100	Ua101	F-M	NA	0.49	0.36	0.60	Known

Sex refers to whether the individual is male (M) or female (F). Hap refers to the mitochondria haplotype (North A = NA, North B = NB, South = S) of both individuals in the pairs (none of the pairs differed). P-O indicates possible parent-offspring dyads determined by identifying pairs that share at least one allele at every locus and additionally in one pair (Ua19 and Ua85) through analysis of the X-chromosome. The two known parent-offspring dyads were confirmed by the presence of at least one shared allele at every locus.

## Conclusions

We present a new panel of 96 SNPs suitable for assaying the Scandinavian brown bear for relatedness and other ecological and evolutionary analyses. Through application of an NGS based RRL approach, we successfully reduced the computational power required to the extent that most analyses were performed on a standard-specification personal computer. This was made possible by eliminating sequences (within the limits of the study) that did not meet strict quality control (eg. inclusion of cut site, questionable quality of putative SNP) and avoiding the often problematic, computationally demanding and error-prone step of sequence assembly through the use of one restriction enzyme. While some applications require a greater number of SNPs, other applications may actually become disadvantaged by too much information. This is likely the case for relatedness studies and thus allowed us the freedom to rapidly decrease the amount of data we analyzed. 

This SNP-chip holds much promise for conservation of the Scandinavian brown bear, particularly for the southern population, which is one of the few relic western European populations. There are many potential uses for this SNP-chip including the use of relatedness estimates to monitor the genetic health, identify mating patterns and reproductive success, and track individual movements. It can also be useful for estimating population size based on individual identification, detecting hybridization events between the northern and southern populations, and confirming paternity in possible multiple paternity events or cases of infanticide. 

## Supporting Information

Table S1 **dbSNP submitted SNP (ss#) numbers and descriptive statistics for autosomal SNPs.**
(DOCX)
