# Supplementary material for: De Novo SNP Discovery in the Scandinavian Brown Bear (Ursus arctos)
Source: PLoS One. 2013 Nov 18;8(11):e81012. doi: 10.1371/journal.pone.0081012 (PMC3832409; doi:10.1371/journal.pone.0081012)
Supplement: Table S1 — dbSNP submitted SNP (ss#) numbers and descriptive statistics for autosomal SNPs. (DOCX) [file pone.0081012.s001.docx]

# Supporting Information

Table S1. dbSNP submitted snp (ss#) numbers and descriptive statistics for autosomal SNPs. Includes minor allele frequency (MAF), expected and observed heterozygosity (H_E_ and H_O_ respectively) and overall F_ST_ values (No. Pops. = 3) for unrelated brown bears (n=50).

|  |  |  |  |  |  |  |  |
| --- | --- | --- | --- | --- | --- | --- | --- |
| SNP Identifier | dbSNP (ss #) | MAF | H_E_ | H_O_ | F_ST_ |  | Notes |
|  |  |  |  |  |  |  |  |
|  |  |  |  |  |  |  |  |
| Ua01y | N/A | 0.00 | 0.00 | 0.00 | N/A |  | Y-chromosome sex determination marker |
| Ua02y | N/A | 0.00 | 0.00 | 0.00 | N/A |  | Y-chromosome sex determination marker |
| Ua03mt | N/A | 0.48 | 0.00 | 0.00 | N/A |  | mtDNA haploid marker |
| Ua04mt | N/A | 0.31 | 0.00 | 0.00 | N/A |  | mtDNA haploid marker |
| Ua05mt | N/A | 0.48 | 0.00 | 0.00 | N/A |  | mtDNA haploid marker |
| Ua07mt | N/A | 0.48 | 0.00 | 0.00 | N/A |  | mtDNA haploid marker |
| Ua101 | 778079577 | 0.47 | 0.50 | 0.38 | -0.02 |  |  |
| Ua102 | 778079578 | 0.44 | 0.49 | 0.44 | 0.23 |  |  |
| Ua104 | 778079579 | 0.27 | 0.39 | 0.34 | 0.25 |  |  |
| Ua105 | 778079580 | 0.45 | 0.50 | 0.42 | 0.02 |  |  |
| Ua111 | 778079581 | 0.42 | 0.49 | 0.44 | 0.06 |  |  |
| Ua112 | 778079582 | 0.35 | 0.46 | 0.42 | 0.09 |  |  |
| Ua114 | 778079583 | 0.48 | 0.50 | 0.40 | 0.16 |  |  |
| Ua115 | 778079584 | 0.50 | 0.50 | 0.52 | 0.02 |  |  |
| Ua116 | 778079585 | 0.42 | 0.49 | 0.44 | 0.15 |  |  |
| Ua118 | 778079586 | 0.48 | 0.50 | 0.44 | 0.06 |  |  |
| Ua119 | 778079587 | 0.44 | 0.49 | 0.48 | 0.08 |  |  |
| Ua120 | 778079588 | 0.28 | 0.40 | 0.36 | -0.03 |  |  |
| Ua125 | 778079589 | 0.46 | 0.50 | 0.48 | 0.03 |  |  |
| Ua127 | 778079590 | 0.33 | 0.44 | 0.42 | 0.11 |  |  |
| Ua128 | 778079591 | 0.37 | 0.47 | 0.42 | 0.11 |  |  |
| Ua129 | 778079592 | 0.47 | 0.50 | 0.58 | 0.03 |  |  |
| Ua131 | 778079593 | 0.34 | 0.45 | 0.32 | 0.17 |  |  |
| Ua133 | 778079594 | 0.42 | 0.49 | 0.52 | -0.02 |  |  |
| Ua134 | 778079595 | 0.40 | 0.48 | 0.44 | 0.16 |  |  |
| Ua136 | 778079596 | 0.32 | 0.44 | 0.32 | 0.36 |  |  |
| Ua141 | 778079597 | 0.36 | 0.46 | 0.32 | 0.20 |  |  |
| Ua145 | 778079598 | 0.45 | 0.50 | 0.46 | 0.23 |  |  |
| Ua147 | 778079599 | 0.38 | 0.47 | 0.56 | 0.01 |  |  |
| Ua150 | 778079600 | 0.44 | 0.49 | 0.48 | -0.02 |  |  |
| Ua156 | 778079601 | 0.38 | 0.47 | 0.40 | 0.05 |  |  |
| Ua159 | 778079602 | 0.35 | 0.46 | 0.54 | 0.03 |  |  |
| Ua160 | 778079603 | 0.35 | 0.46 | 0.46 | 0.10 |  |  |
| Ua162 | 778079604 | 0.27 | 0.39 | 0.30 | -0.04 |  | Significant for HWD in South population (n=23; p-value=0.0345) |
| Ua163 | 778079605 | 0.33 | 0.44 | 0.34 | 0.34 |  | Linked with Ua214 (D’=0.9996) |
| Ua164 | 778079606 | 0.48 | 0.50 | 0.52 | 0.06 |  |  |
| Ua165 | 778079607 | 0.39 | 0.48 | 0.50 | 0.13 |  |  |
| Ua166 | 778079608 | 0.38 | 0.47 | 0.36 | 0.22 |  | Significant for HWD in North population (n=16; p-value=0.0159) |
| Ua168 | 778079609 | 0.46 | 0.50 | 0.52 | 0.06 |  |  |
| Ua169 | 778079610 | 0.38 | 0.47 | 0.44 | 0.00 |  |  |
| Ua170 | 778079611 | 0.25 | 0.38 | 0.34 | -0.03 |  | Significant for HWD in North population (n=16; p-value=0.0467) |
| Ua171 | 778079612 | 0.46 | 0.50 | 0.40 | 0.10 |  | Linked with Ua227 (D’=0.9411) |
| Ua172 | 778079613 | 0.43 | 0.49 | 0.42 | 0.03 |  |  |
| Ua175 | 778079614 | 0.33 | 0.44 | 0.42 | 0.12 |  |  |
| Ua176 | 778079615 | 0.44 | 0.49 | 0.56 | -0.02 |  |  |
| Ua177 | 778079616 | 0.37 | 0.47 | 0.42 | -0.01 |  |  |
| Ua179 | 778079617 | 0.46 | 0.50 | 0.56 | 0.01 |  | Significant for HWD in North population (n=16; p-value=0.0145) |
| Ua180 | 778079618 | 0.39 | 0.48 | 0.46 | 0.05 |  |  |
| Ua181 | 778079619 | 0.26 | 0.38 | 0.32 | 0.11 |  |  |
| Ua183 | 778079620 | 0.40 | 0.48 | 0.56 | 0.03 |  |  |
| Ua184 | 778079621 | 0.42 | 0.49 | 0.52 | 0.12 |  |  |
| Ua186 | 778079622 | 0.34 | 0.45 | 0.40 | 0.16 |  |  |
| Ua189 | 778079623 | 0.38 | 0.47 | 0.48 | 0.09 |  |  |
| Ua191 | 778079624 | 0.41 | 0.48 | 0.54 | -0.02 |  |  |
| Ua193 | 778079625 | 0.44 | 0.49 | 0.52 | 0.20 |  |  |
| Ua195 | 778079626 | 0.32 | 0.44 | 0.32 | 0.18 |  | Significant for HWD in South population (n=23; p-value=0.0217) |
| Ua196 | 778079627 | N/A | N/A | N/A | N/A |  | X-chromosome SNP |
| Ua199 | 778079628 | 0.49 | 0.50 | 0.50 | 0.01 |  |  |
| Ua200 | 778079629 | 0.30 | 0.42 | 0.40 | -0.03 |  |  |
| Ua201 | 778079630 | 0.26 | 0.38 | 0.36 | 0.14 |  |  |
| Ua202 | 778079631 | 0.39 | 0.48 | 0.50 | 0.06 |  |  |
| Ua203 | 778079632 | 0.35 | 0.46 | 0.46 | -0.02 |  |  |
| Ua204 | 778079633 | 0.39 | 0.48 | 0.42 | -0.01 |  |  |
| Ua205 | 778079634 | 0.44 | 0.49 | 0.36 | -0.03 |  | Significant for HWD in South population (n= 23; p-value=0.0087) |
| Ua206 | 778079635 | 0.42 | 0.49 | 0.48 | -0.02 |  |  |
| Ua207 | 778079636 | 0.29 | 0.41 | 0.38 | 0.20 |  |  |
| Ua209 | 778079637 | 0.46 | 0.50 | 0.48 | 0.08 |  |  |
| Ua211 | 778079638 | 0.43 | 0.49 | 0.46 | 0.20 |  |  |
| Ua212 | 778079639 | 0.47 | 0.50 | 0.46 | -0.01 |  |  |
| Ua213 | 778079640 | 0.29 | 0.41 | 0.42 | 0.15 |  |  |
| Ua214 | 778079641 | 0.32 | 0.44 | 0.40 | 0.04 |  | Linked with Ua163 |
| Ua217 | 778079642 | 0.31 | 0.43 | 0.42 | 0.05 |  |  |
| Ua218 | 778079643 | 0.44 | 0.49 | 0.48 | 0.26 |  |  |
| Ua219 | 778079644 | 0.41 | 0.48 | 0.50 | 0.04 |  |  |
| Ua220 | 778079645 | 0.36 | 0.46 | 0.48 | 0.03 |  |  |
| Ua221 | 778079646 | 0.34 | 0.45 | 0.40 | 0.32 |  |  |
| Ua222 | 778079647 | 0.47 | 0.50 | 0.54 | -0.01 |  |  |
| Ua223 | 778079648 | 0.44 | 0.49 | 0.48 | -0.01 |  |  |
| Ua225 | 778079649 | 0.44 | 0.49 | 0.44 | 0.02 |  |  |
| Ua226 | 778079650 | 0.37 | 0.47 | 0.42 | -0.03 |  |  |
| Ua227 | 778079651 | 0.34 | 0.45 | 0.44 | -0.02 |  | Linked with Ua171 |
| Ua228 | 778079652 | 0.36 | 0.46 | 0.48 | 0.15 |  |  |
| Ua230 | 778079653 | 0.43 | 0.49 | 0.46 | -0.03 |  |  |
| Ua231 | 778079654 | 0.31 | 0.43 | 0.38 | -0.02 |  | Significant for HWD in North population (n=16; p-value=0.0162) |
| Ua234 | 778079655 | 0.33 | 0.44 | 0.46 | -0.02 |  |  |
| Ua236 | 778079656 | N/A | N/A | N/A | N/A |  | X-chromosome SNP |
| Ua237 | 778079657 | 0.46 | 0.50 | 0.44 | 0.00 |  |  |
| Ua239 | 778079658 | 0.39 | 0.48 | 0.50 | 0.16 |  |  |
| Ua240 | 778079659 | 0.35 | 0.46 | 0.42 | -0.02 |  |  |
| Ua241 | 778079660 | 0.32 | 0.44 | 0.36 | 0.04 |  |  |
| Ua244 | 778079661 | 0.39 | 0.48 | 0.42 | -0.04 |  |  |
| Ua245 | 778079662 | 0.36 | 0.46 | 0.44 | -0.01 |  |  |
| Ua248 | 778079663 | N/A | N/A | N/A | N/A |  | X-chromosome SNP |
| Ua250 | 778079664 | 0.29 | 0.41 | 0.26 | 0.40 |  |  |
| Ua251 | 778079665 | 0.42 | 0.49 | 0.56 | 0.00 |  |  |
| Ua253 | 778079666 | 0.37 | 0.47 | 0.46 | 0.00 |  |  |
|  |  |  |  |  |  |  |  |
